# Footing the bill: the introduction of Medicare Benefits Schedule rebates for podiatry services in Australia

**DOI:** 10.1186/1757-1146-2-36

**Published:** 2009-12-07

**Authors:** Anthony J Short

**Affiliations:** 1 School of Public Health, Queensland University of Technology, Brisbane, Australia

## Abstract

The introduction of Medicare Benefits Schedule items for allied health professionals in 2004 was a pivotal event in the public funding of non-medical primary care services. This commentary seeks to provide supplementary discussion of the article by Menz (Utilisation of podiatry services in Australia under the Medicare Enhanced Primary Care program, 2004-2008 *Journal of Foot and Ankle Research *2009, **2:**30), by placing these findings within the context of the podiatry profession, clinical decision making and the broader health workforce and government policy.

## Introduction

The Enhanced Primary Care (EPC) program was introduced in 1999 as a range of measures targeted at primary care to improve the quality of chronic disease management. Under the Howard Government in 2004, the then Minister for Health and Ageing, the Honorable Tony Abbott, modified the EPC program under the *Strengthening Medicare *initiative to provide limited access to allied health professional (AHP) services under the Medicare Benefits Schedule (MBS). New MBS item numbers were introduced for AHPs, such as podiatrists, managing chronic disease in primary health care settings where a General Practitioner (GP) Management Plan (GPMP) and Team Care Arrangements (TCA), had been developed by a patients' GP. These acronyms alone may well have alerted the astute health care observer to the tidal wave of paperwork and form-filling that was to begin permeating widely through Australian primary care [1].

Speaking at the time of the introduction of the new MBS items for AHPs, the Minister announced: "This model confirms the holistic role of GPs to manage the health needs of their patients. It will mean GPs have more flexibility and increased options to ensure their patients can access a range of treatment options. This model aims to limit red tape for GPs and ensure that chronically ill patients get the allied health services they need" [2]. However a reduction in red tape for GPs was certainly not a feature of the evolving EPC program.

The recent article by Menz, which has addressed the utilisation of the MBS item for podiatry services over 2004-2008 under the EPC program [3], is possibly the first podiatry profession-specific examination of the MBS dataset related to the allied health EPC items. This significant work provides an insight into the economic and demographic uptake of a solitary item number, but an item number nevertheless that has served as a revolutionary (or evolutionary) milestone in the recognition and acceptance of podiatrists' roles within Australian primary health care.

## Podiatry within the Enhanced Primary Care Program

Prior to 2004, AHPs could not provide any rebatable services for patients under the MBS. Health consumers in Australia seeking AHP services were only able to seek subsidised care if they were seen by a state-funded public sector AHP provider, or had suitable private health insurance or third party coverage under Veterans' Affairs or WorkCover arrangements. Alternatively, patients needed to be self-funded. The introduction of the allied health items under the EPC program allowed only those patients with a deemed 'chronic condition', following GP assessment and planning, to be eligible for accessing a small number of MBS rebatable AHP consultations per year.

According to Menz [3], the first five years of the utilisation of MBS (Item 10962) for podiatry represented over 1.3 million consultations, only marginally less than the item for physiotherapists - the AHP group providing the largest number of EPC consultations. Considering the relative sizes of these professional groups, an interesting extension to this study would have been to examine the number of EPC services per health professional. Australian health workforce data cited by Menz inferred that there were approximately 14,300 practicing physiotherapists, versus 1,800 podiatrists [4]. Therefore, as almost an equivalent number of consultations were provided by physiotherapists and podiatrists under the EPC program over this period, it could be approximated that podiatrists were *individually *providing 6-7 times more EPC services than physiotherapists. This represents a large portion of clinical loading that must be stretching capacity within the Australian podiatry labour market.

Menz has demonstrated that between 2004-2008, the total MBS expenditure on podiatry services for item 10962 was close to $AUD63million, with substantial growth in the number of services provided both in absolute terms, and relative to those enrolled within Medicare. This is unquestionably a substantial figure relative to the size of the profession, and requires that this taxpayer investment be more thoroughly examined. Research should now be undertaken to look specifically at exactly what types of services podiatrists and other AHPs are providing to Australian communities within primary health care, if this service model represents best practice, or is an improvement in access and health outcomes over previous non-shared care arrangements. The difficulty in doing this will be linking datasets held by GPs in practice, and data administered under the Pharmaceutical Benefits Scheme (PBS) or Veterans' Affairs.

The greatest limitation of the policy underpinning the EPC program is that it is constrained to five consultations per patient shared across AHPs nominated by the patient's GP. It would be difficult to argue by any test that this funding arrangement would represent best practice (or even minimum standards) for most chronic disease management by AHPs. Furthermore, the limitation of simply funding a 'consultation' fee and none of the associated services and supplies with management means that these costs can only be borne by the patient. It has been suggested that clinical outcomes may be adversely affected by adhering to the services allocated under EPC funding provided, and that inequities maintained where socioeconomic status affects the ability of patients to pay. This creates a situation where AHPs are forced to develop treatment strategies which are at variance with recommended best practice [5].

In this light, the payments provided to GPs as a precursor to referral to AHPs under the EPC program are worthy of some consideration. As at 2009, the requirement for the (compulsory) preparation of an initial GPMP and documenting TCAs (items 721 and 723), prior to EPC allied health referral, represented $234.15 per patient in health spending to GPs. These GP rebates together represent almost as much as the total available funding pool for service provision by AHPs per annum. TCAs have already been questioned by others [1,6] as lacking an evidentiary basis and requiring all team members to agree on a proposed management plan, despite conventional referral processes relying on professional judgement to determine appropriate pathways. Considering that referral of patients from GPs to medical specialists under the MBS does not require such burdensome and costly administrative processes, one must query why two quite similar referral processes are treated so differently. Moreover, AHPs are front line healthcare practitioners that traditionally provide clinical management for patients without a medical referral (though still in a collaborative manner), and Menz has rightly questioned the need for this 'gatekeeper' role into the future. Removing the requirement for a TCA to be in place has been recommended as one means of simplifying the process and producing savings [7].

With the rhetoric of health planning moving towards a more patient-centred health care system, it is disappointing that no studies have yet been undertaken to evaluate the impact of the EPC and AHP items on patient outcomes. GPs and AHPs themselves have often been left confused and disorientated by the complex nature of seeing patients under the EPC program, with the substantial bureaucratic requirements associated with it. It would be reasonable to assume that patients, with even less understanding of the complexities of navigating the health system, may be even more frustrated with the process of simply getting a timely referral to an appropriate AHP provider.

## Discussion

Chronic disease, by its nature, is often complex, and associated with a range of comorbidities that can adversely affect clinical outcomes. Although the dataset examined by Menz [3] cannot possibly provide advice on the relevant diagnoses leading to the referral of patients to podiatrists and AHPs under the EPC program, it is reasonable to speculate that diabetes (and its foot complications), is the chronic disease that podiatrists would most likely encounter in a primary health care setting. It is unavoidable that the limited number of AHP services accessible under the EPC program means that patients, their GPs, and eventually individual podiatrists are faced with making difficult ethical and economic choices under this framework.

For example, a foot ulcer secondary to diabetes-induced peripheral neuropathy is well described within the scientific literature as a costly and labour intensive clinical scenario to manage. Various procedures and treatments are required to provide a successful outcome and avoid more serious and costly complications and hospitalisation costs. The frequency and duration of care may be high during the acute stages, followed by episodic monitoring to prevent recurrences. The many facets of examination (e.g. Doppler ultrasound) and treatment (e.g. surgical debridement, specialised wound care products and mechanical offloading) mean that allocated podiatry visits under the EPC program can be exhausted swiftly, and associated costs for non-covered services and further ongoing care are either borne by the patient, or not provided at all if alternative public services are unavailable. The ethical dilemma of developing a treatment plan in situations where patients can only be seen under such tight and rationed funding criteria (i.e. where no alternative public services are available), whilst attempting to provide best practice management for a complex condition, would be generally unfamiliar (or unacceptable) to most medical practitioners. However, this view must be gracefully tempered with the obvious reality that no MBS funding was available at all for AHP services prior to 2004.

The 'one size fits all' approach to funding allied health services under the EPC program might have been a convenient solution to avoiding a raft of new and differing item numbers for AHPs in the MBS, but it has created a situation that rebates all allied services at the same level, regardless of the complexity, costs or resources required to deliver the service. Further reform of the EPC items must urgently take these variables into account to reflect the inherent core differences in services provided by different AHPs, as is already done by the Department of Veterans' Affairs.

Increasing the overall number of consultations to AHPs has been suggested by Allied Health Professions Australia [7]. Their proposal to fund AHP services following the same model as *the Better access to psychiatrists, psychologists and general practitioners through the Medicare Benefits Scheme *initiative recommends up to a maximum 18 consultations per year, with a continuation of the gatekeeper role for GPs. They also recommend differing tiers of rebates to move away from a single level of rebate for all AHP services. Though this would no doubt be popular with clinicians and patients, it will be difficult to fund without impacting on the overall allocated health budget.

The recently released report and draft of the *National Primary Health Care Strategy *[8,9] has clearly identified that reform of Australian primary health care is needed. It has raised workforce pressures, equity of access concerns and the trend towards community-based (rather than hospital-based) care as being major drivers of this process. Given that the EPC program is just one part of the unwieldy and confusing spectrum of federal, state and specific targeted funding initiatives, it is timely that cohesive and less complex options are being considered. With the Australian workforce of AHPs being larger in size than the medical practitioner workforce [9], the oft-repeated calls for broader scope of practice funding for non-medical practitioners under the MBS are growing. The pending introduction of proposed nurse practitioner and midwife rebates under the MBS and PBS may yet prove to be a template for further integration of AHPs within mainstream Commonwealth primary care funding. The impetus to widen the focus of health workforce reform beyond 'doctors and nurses', by including high-demand health professions such as podiatry into more mainstream health funding models, could well be the eventual legacy of the EPC program.

However, one final question may be worth asking as these strategies are developed. Why does the MBS not fund acute care services by podiatrists and AHPs?

## Conclusion

The introduction of allied health items under the Enhanced Primary Care program has no doubt been beneficial to improving access to allied health services for people with chronic disease. The utilisation of podiatry services under the EPC program, as described by Menz [3], has highlighted the popularity and demand for podiatrists. Given the increasing demand over time observed within this report, it is likely that there will be continued growth for podiatry services, also in line with forecasts for population growth and the trend towards an ageing society, which in turn has broader implications for workforce planning and training.

There are substantial opportunities for further restructuring and refinement of the funding of AHP services under the MBS, and the pending reforms recommended by the *National Health and Hospitals Reform Commission *and *National Primary Health Care Strategy*, if implemented in line with available evidence, should produce improved access and outcomes for Australians.

## Abbreviations

EPC: Enhanced Primary Care; MBS: Medicare Benefits Schedule; TCA: Team Care Arrangements; GPMP: General Practitioner Management Plan; AHP: Allied Health Professional; GP: General Practitioner.

## Competing interests

The author declares that they have no competing interests.

## Author's information

Anthony Short is a visiting lecturer in radiology and podiatric surgery at the Queensland University of Technology, School of Public Health. He is a visiting podiatrist to the Queensland Diabetes Centre, and has been involved in the provision of primary care services to local Divisions of General Practice. He holds memberships with the Australasian Podiatry Association (Qld), the Australasian College of Podiatric Surgeons, and the Australian Wound Care Association (Qld). He has also worked in private practice in Brisbane for 12 years.

## Acknowledgements

Thanks are given to Dr Sue Wicker, CEO of the Australasian Podiatry Council, for reviewing the initial draft and providing valuable comments and advice.
